# AGE DIFFERENCES IN EMOTION REGULATION AND EXECUTIVE FUNCTION

**DOI:** 10.1093/geroni/igad104.3169

**Published:** 2023-12-21

**Authors:** Wei Xing Toh, Hwajin Yang

**Affiliations:** Nanyang Technological University, Singapore, Singapore; Singapore Management University, Singapore, Not Applicable, Singapore

## Abstract

Age-related improvements in emotion regulation (ER) are thought to account for the relatively healthy levels of well-being observed in late adulthood. However, extant laboratory-based evidence on age differences in the effective implementation of ER strategies―such as reappraisal (i.e., reframing the meaning of an emotion-eliciting event) and distraction (i.e., redirecting attention away from emotional to non-emotional information)―remains equivocal (Isaacowitz, 2022). Moreover, developmental differences in ER monitoring (i.e., adjusting regulatory approach as needed, such as maintaining or switching strategies), and factors that underpin such age-related differences, are poorly understood. Therefore, we investigated how younger and older adults (N = 335) differ in the implementation and monitoring aspects of ER, and how executive function (EF)―a collection of higher-order control operations that underlie ER processes (Pruessner et al., 2020; Schmeichel & Tang, 2015)―could explain such age-related differences in ER. Using a comprehensive battery of nine EF and two ER tasks, results indicated age-related decline for the implementation of reappraisal but not distraction. For monitoring, advancing age was associated with a less flexible pattern of strategy maintenance and switching (of reappraisal and distraction) across high- and low-intensity emotional contexts. Importantly, these age-related differences in ER implementation and monitoring were significantly mediated by declines in EF (βs = .25‒.29, ps < .007), thereby alluding to the role of EF as a potential mechanism. Our findings suggest that certain ER processes are more cognitively demanding than others, and that EF could be a viable target of intervention to improve such ER processes in late adulthood.

